# Supplementary material for: Effect of Fortified Inoculation with Indigenous Lactobacillus brevis on Solid-State Fermentation of Light-Flavor Baijiu
Source: Foods. 2023 Nov 21;12(23):4198. doi: 10.3390/foods12234198 (PMC10706162; doi:10.3390/foods12234198)
Supplement: Supplementary file 1 [file foods-12-04198-s001.zip › foods-2594543-supplementary.pdf]

Table S1. Primers and real-time quantitative PCR conditions used

PCR target(gene)	Primer name	Primer sequence	Amplicon size	PCR cycling conditions	Reference
Total bacterial population (16S)	P1	CCTACGGGAGGCAGCAG	196 bp	94°C for 10 s, then 40 cycles of 94°C for 5 s, 58°C for15 s, 72°C for 15 s, and finally one cycle of 72°C for 5 min.	[38]
	P2	ATTACCGCGGCTGCTGG			
Total fungal population (18S)	F1	GCGGTAATTCCAGCTCCAATAG	151 bp	one cycle of 94°C for 10 s, then 45 cycles of 94°C for 5 s, 56°C for 15 s, 72°C for 15 s, and finally one cycle of 72°C for 5 min.	[38]
	F2	GCCACAAGGACTCAAGGTTAG			
Lactobacillus brevis (16S)	Lbrev F	TGCACTGATTTCAACAATGAAG	160 bp	2 min at 98°C followed by 35 cycles of 5 s at 98°C and 30 s at 56.5°C.	[39]
	Lbrev R	CCAGAAGTGATAGCCGAAGC			
Lactobacillus (16S)	F-lac	GCAGCAGTAGGGAATCTTCCA	349 bp	95°C for 30 s; 40 cycles of 95°C for 10 s and 62°C for 30 s.	[40]
	R-lac	GCATTYCACCGCTACACATG			
Saccharomyces (ITS1)	SC1	GAAAACTCCACAGTGTGTTG	124 bp	5 min at 98°C followed by 40 cycles of 15 s at 98°C, 30 s at 63°C, and 30 s at 72°C.	[41]
	SC2	GCTTAAGTGCGCGGTCTTG			

Table S2 Lactic acid bacteria isolated in fermented grains

Strain ID	Species	Related GenBank sequence	Identity (10%)
Lbu1	*Lactobacillus buchneri*	AB205055	100
Lbu2	*Lactobacillus buchneri*	AB205055	100
Lbu3	*Lactobacillus buchneri*	LC094429	99
Lbu4	*Lactobacillus buchneri*	HM058334.1	100
Lbu5	*Lactobacillus buchneri*	LC094429	99
Lbr6	*Lactobacillus brevis*	DQ268866	100
Lbr7	*Lactobacillus brevis*	KT780303	99
Lbr8	*Lactobacillus brevis*	DQ268866	100
Lbr9	*Lactobacillus brevis*	DQ268866	100
Lbr11	*Lactobacillus brevis*	DQ268866	100
Lbr11	*Lactobacillus brevis*	DQ268866	100
Lbr12	*Lactobacillus brevis*	DQ268866	99
Lbr13	*Lactobacillus brevis*	DQ268866	100
Lbr14	*Lactobacillus brevis*	AP012167	99
Lbr15	*Lactobacillus brevis*	AP012167	99
Lbr16	*Lactobacillus brevis*	LC062086	99
Lbr17	*Lactobacillus brevis*	KT780303	100
Lbr18	*Lactobacillus brevis*	DQ268866	99
Lbr19	*Lactobacillus brevis*	DQ268866	99
Lbr20	*Lactobacillus brevis*	KU315055	100
Lbr21	*Lactobacillus brevis*	DQ268866	99
Lbr22	*Lactobacillus brevis*	DQ268866	99
Lbr23	*Lactobacillus brevis*	DQ268866	100
Lbr24	*Lactobacillus brevis*	DQ268866	100
Lbr25	*Lactobacillus brevis*	DQ268866	100
Lbr26	*Lactobacillus brevis*	DQ268866	100
Lca27	*Lactobacillus casei*	HM058794	99
Lca28	*Lactobacillus casei*	HM058896	100
Lca29	*Lactobacillus casei*	HM058896.1	100
Lca30	*Lactobacillus casei*	HM058896.1	100
Lca31	*Lactobacillus casei*	HM058839.1	100
Lpa32	*Lactobacillus paracasei*	KU315090	100
Lpa33	*Lactobacillus paracasei*	KT962976	100
Lpa34	*Lactobacillus paracasei*	FM878598	99
Lpa35	*Lactobacillus paracasei*	KU315079.1	99

Lpa36	*Lactobacillus paracasei*	KU315090.1	100
Lhi37	*Lactobacillus hilgardii*	FM878600	100
Lhi38	*Lactobacillus hilgardii*	LC064898	99
Lhi39	*Lactobacillus hilgardii*	FM878600	100
Lhi40	*Lactobacillus hilgardii*	AB262962	99
Lhi41	*Lactobacillus hilgardii*	AB262962	99
Lhi42	*Lactobacillus hilgardii*	AB262962	100
Lhi43	*Lactobacillus hilgardii*	AB262962	100
Lhi44	*Lactobacillus hilgardii*	AB262962	100
Lhi45	*Lactobacillus hilgardii*	AB262962	99
Lhi46	*Lactobacillus hilgardii*	LC064898	100
Lhi47	*Lactobacillus hilgardii*	LC064898	100
Lhi48	*Lactobacillus hilgardii*	LC064898	100
Lhi49	*Lactobacillus hilgardii*	AB262962	99
Lhi50	*Lactobacillus hilgardii*	FM878600	100

Table S3 Yeasts isolated in fermented grains

Strain ID	Species	Related GenBank sequence	Identity (10%)
Pf1	*Pichia fermentans*	KC494719.1	100
Ho2	*Hanseniaspora osmophila*	GU080047.1	99
Ho3	*Hanseniaspora osmophila*	GU080047.1	99
Ho4	*Hanseniaspora osmophila*	JQ512840.1	99
Sf5	*Saccharomycopsis fibuligera*	CP012809.1	100
Sf6	*Saccharomycopsis fibuligera*	CP012809.1	100
Sc7	*Saccharomyces cerevisiae*	EF192587.1	99
Sc8	*Saccharomyces cerevisiae*	KX119943.1	99
Sc9	*Saccharomyces cerevisiae*	HM191652.1	99
Sc10	*Saccharomyces cerevisiae*	HM191649.1	100
Sc11	*Saccharomyces cerevisiae*	KU862641.1	100
Sc12	*Saccharomyces cerevisiae*	GU080048.1	100
Sc13	*Saccharomyces cerevisiae*	HM191661.1	99
Sc14	*Saccharomyces cerevisiae*	KX428530.1	99
Sc15	*Saccharomyces cerevisiae*	HM107800.1	99
Sc16	*Saccharomyces cerevisiae*	EU884435.1	100
Sc17	*Saccharomyces cerevisiae*	HM191661.1	100
Sc18	*Saccharomyces cerevisiae*	KY109244.1	99
Sc19	*Saccharomyces cerevisiae*	KX428529.1	99
Sc20	*Saccharomyces cerevisiae*	HM101472.1	99
Sc21	*Saccharomyces cerevisiae*	HM107783.1	99
Sc22	*Saccharomyces cerevisiae*	HM191648.1	100
Sc23	*Saccharomyces cerevisiae*	KX428530.1	99
Sc24	*Saccharomyces cerevisiae*	KM527244.1	99
Sc25	*Saccharomyces cerevisiae*	KY109393.1	100
Wa26	*Wickerhamomyces anomalus*	KY110104.1	99

Reference

38. Xu W, Huang Z Y, Zhang X J, Li Q, Lu Z M, Shi J S, Xu Z H, Ma Y H. Monitoring the microbial community during solid-state acetic acid fermentation of Zhenjiang aromatic vinegar[J]. Food Microbiology, **2011**, 28(6): 1175-1181.

39. Duniere L, Jin L, Smiley B, Qi M, Rutherford W, Wang Y, Mcallister T. Impact of adding Saccharomyces strains on fermentation, aerobic stability, nutritive value, and select lactobacilli populations in corn silage[J]. Journal of Animal Science, **2015**, 93(5): 82-87.

40. Wu S H, Xu S J, Chen X, Sun H S, Hu M L, Bai Z H, Zhuang G Q, Zhuang X L. Bacterial communities changes during food waste spoilage[J]. Scientific Reports, **2018**, 8(1): 20-29.
41. Katharina Z, Olivier C, Patrick M L, Joana C, Aline L, Isabelle M P. Characterization of the yeast ecosystem in grape must and wine using real-time PCR[J]. Food Microbiology, **2010**, 27(5): 559-567
